# An Experimental and Computational Study of the High-Velocity Impact of Low-Density Aluminum Foam

**DOI:** 10.3390/ma13081949

**Published:** 2020-04-21

**Authors:** Matej Borovinšek, Matej Vesenjak, Kazuyuki Hokamoto, Zoran Ren

**Affiliations:** 1 Faculty of Mechanical Engineering, University of Maribor, 2000 Maribor, Slovenia; matej.vesenjak@um.si (M.V.); zoran.ren@um.si (Z.R.); 2 Institute of Pulsed Power Science, Kumamoto University, 2-39-1 Kurokami, Kumamoto City 860-8555, Japan; hokamoto@mech.kumamoto-u.ac.jp

**Keywords:** low-density aluminum foam, Taylor impact test, computer simulations, finite element method

## Abstract

The study presents the results of an experimental and computational study of the high-velocity impact of low-density aluminum foam into a rigid wall. It is shown that the aluminum foam samples deformed before hitting the rigid wall because of the high inertial forces during the acceleration. During the impact, the samples deformed only in the region contacting the rigid wall due to the high impact velocity; the inertial effects dominated the deformation process. However, the engineering stress–strain relationship retains its typical plateau shape until the densification strain. The experimental tests were successfully reproduced with parametric computer simulations using the LS-DYNA explicit finite element code. A unique computational lattice-type model was used, which can reproduce the randomness of the irregular, open-cell structure of aluminum foams. Parametric computer simulations of twenty different aluminum foam sample models with randomly generated irregular lattice structures were carried out at different acceleration levels to obtain representative statistical results. The high strain-rate sensitivity of low-density aluminum foam was also observed. A comparison of experimental and computational results during aluminum foam sample impact shows very similar deformation behavior. The computational model correctly represents the real impact conditions of low-density aluminum foam and can be recommended for use in similar high-velocity impact investigations.

## 1. Introduction

Lightweight materials have unique physical and mechanical properties, such as low density and high specific stiffness, and are being increasingly used in modern industrial applications [1]. Metallic cellular materials are very suitable for use in impact-energy absorbers, which sustain deformations under high strain-rates [2,3]. However, this type of material has become a subject of investigation only in the past decade, and there are only a limited number of research results available regarding the strain-rate’s influences on the deformation mechanisms of metallic cellular materials.

Rinde and Hoge [4] have studied the strain-rate effects on the compressive stiffness of Styrofoam (polystyrene) at room temperature and discovered that its stiffness increases only slightly with increasing strain-rate. Dung et al. [5] studied the strain-rate effects on polyvinyl chloride foams and confirmed that the strain-rate dependency is becoming more significant with the increase of foam density. Mukai et al. [6] comparatively studied the strain-rate effects on the polystyrene and closed-cell aluminum foam (ALPORAS) with the same relative density *ρ*_r_. They proved that the plateau stresses of the aluminum foam are much more sensitive to an increase in strain-rate than the polystyrene. Pawel et al. [7] successfully used ALPORAS foam as a filler of the magnesium alloy crash-boxes, demonstrating an increase of 53% in its crashworthiness. Mukai et al. [8] investigated the strain-rate dependency of open-cell magnesium foams (AZ91) under compressive loading. They discovered that the mechanical energy absorption is approximately 100% higher at a strain rate of 1400 s^−1^ in relation to the quasi-static loading conditions. They proved that the amount of absorbed mechanical work heavily depends on the applied strain rate since. Christ et al. [9] investigated the compressive mechanical properties of closed-cell cellular materials at strain rates from 2 × 10^−4^ to 2 s^−1^. They have demonstrated that closed-cell materials change their deformation behaviors with the strain-rate increase due to micro-inertial effects, which increase the apparent material stiffness. The increased stiffness contributes to an increase in the absorbed mechanical energy [10]. Wang et al. [11] and Duarte et al. [12] also made similar conclusions in their studies.

The structures of cellular materials strongly influence their properties [13,14,15]. They are often highly irregular, which limits the reproducibility of experimental investigations. The use of experimental methods to study and understand their behavior is often also time-consuming and costly [16,17,18]. Studying internal deformation mechanisms of cellular materials thoroughly or measuring specific physical properties during experimental testing is very difficult and often even impossible. Computer simulations based on relevant mathematical models have recently become a desirable alternative with which to advance the understanding of a cellular material’s behavior under different loading conditions. Vesenjak et al. [19] performed a computational study of dynamic low-density metal foam behavior, considering only the representative volume element of the sample.

Some engineering computer simulation systems utilize homogenized constitutive models of cellular materials. However, their usefulness in dynamic simulations is limited since they do not account for the geometric irregularity and strain-rate effects appropriately. This was recently overcome with the development of a new computational lattice-type model of an irregular, open-cell material [20]. The model properly accounts for the effects of structural irregularity and different strain-rates on the mechanical behaviors of open-cell materials subjected to large deformations. The quasi-static and dynamic uniaxial compressive experiments of low-density aluminum open-cell foams were used to validate the model successfully [19]. 

The Taylor impact tests of the aluminum foam samples were carried out in this paper as a follow-on study to understand the impact deformation phenomenon better and to validate the lattice-type model of aluminum foams at higher impact velocities as well. Understanding of the strain rate sensitivity and energy absorption of metal foams is crucial for the crash absorbers’ development in the aircraft, spacecraft, automotive and military industries, wherein high efficiency combined with low weight is a fundamental prerequisite. Computational simulations using a validated computational model can help with finding the optimal design for virtually any type of loading scenario, thereby significantly advancing the crash absorber’s development.

## 2. Experimental Setup 

**Figure 1:**
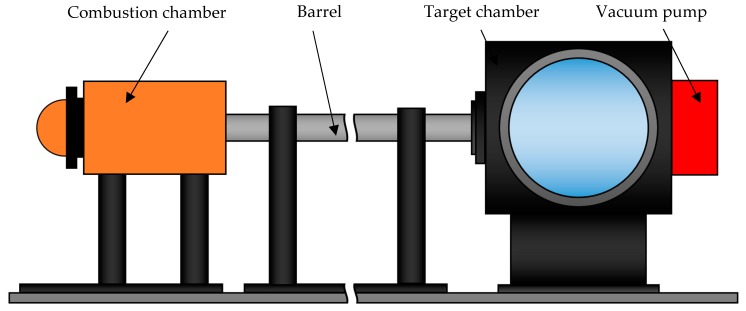
A schematic representation of the Taylor impact test device.

The Taylor impact test was applied to study the behavior of low-density open-cell aluminum foam at higher strain-rates [21,22]. A foam specimen was accelerated to the desired velocity by a gas gun and then impacted into a rigid wall. The Taylor impact tests were carried out at the Institute of Pulsed Power Science, Kumamoto University, Japan. The testing device consists of a combustion chamber, a 3 m long barrel, and a target chamber, Figure 1 [23]. The target chamber allows for optical observations of the experiments through reinforced windows. The air is removed from the target chamber and barrel to a near-vacuum pressure with the vacuum pump, minimizing air resistance’s influence on the projectile. A special diaphragm placed between the combustion chamber and the barrel is used to control the release of combustion gases in the chamber by its rupture at a specified pressure. A steel plate with a diameter of 90 mm and a thickness of 2.5 mm was used for that purpose in this study. The projectile is positioned in the barrel so that it is in contact with the diaphragm.

**Figure 2:**
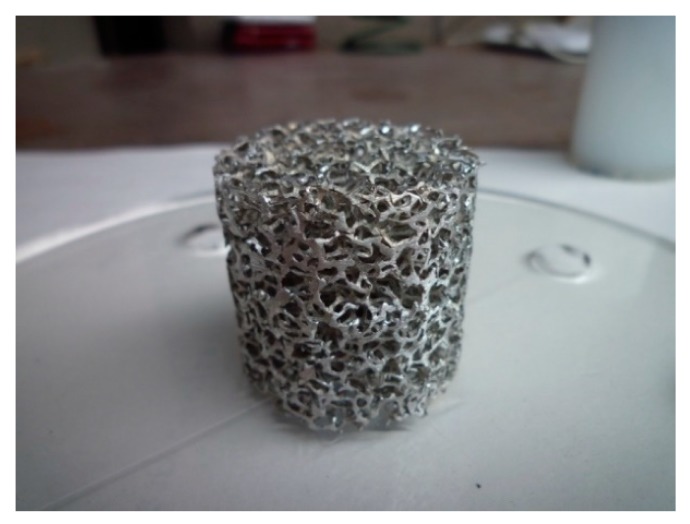
Cylindrical foam sample (d = 35 mm; h = 35 mm).

**Figure 3:**
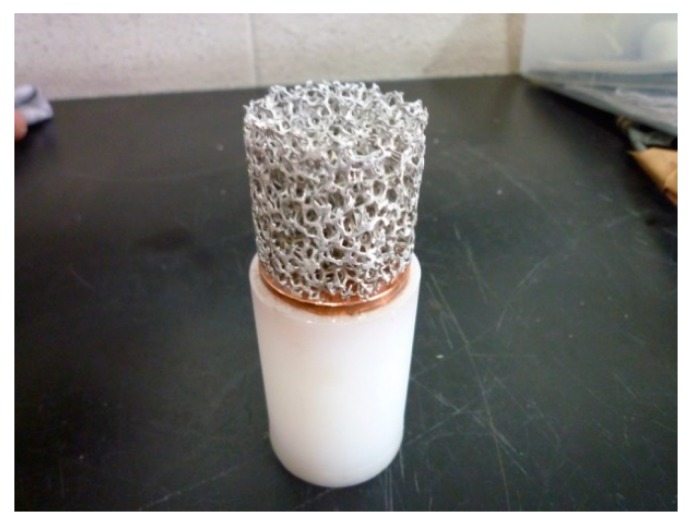
Sample attached to the copper plate and the sabot.

The projectile comprised a cylindrically shaped aluminum foam sample (Figure 2) and the copper plate inserted into a plastic sabot (Figure 3). The samples were made from the low-density, open-cell aluminum foam produced by m-pore GmbH from pure aluminum EN AW-1070 (99.7% purity). The relative density of samples was 6.1% (93.9% porosity) with a cell size of 10 PPI and the mean cell diameter of 3.8 mm. The sample diameter and height were 35 × 35 mm, with a total mass of 5.60 g. The copper plate with a thickness of 5 mm served as the impact velocity regulator. The total projectile mass was 114.65 g.

The nitrocellulose-based explosive of 8 g was used in this study as the accelerant, together with a small amount of the black powder (2 g) used for the ignition, activated by an electric spark. This combination accelerated the above-described projectile to the final muzzle velocity of 400 m/s. More detailed information about the experimental setup, testing and results is given in Tanaka et al. [23].

**Figure 4:**
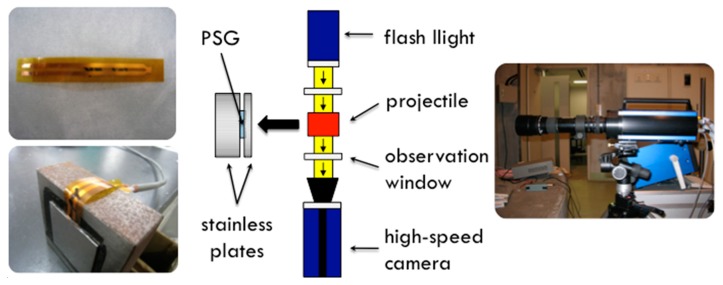
The shadowgraph and pressure measurement (PSG) experimental setup.

Due to extensive fracturing of the aluminum foam at high impact velocities, the dynamic material properties of the foam could not be determined from the projectile deformation after the impact by using the analytical solution proposed initially by Taylor [21]. Instead, an optical observation with a shadowgraph method was used to observe the deformation process of aluminum foam samples. The shadowgraph method, also known as the direct projection technique, is based on recording a light shadow projection on a camera (Figure 4). The camera in front of the object records only light passing by or through the object, depicting the non-translucent objects as shadows on the taken image. The Hyper Vision HPV-1 high-speed camera, produced by SHIMADZU Corporation, was used for shadowgraph observations of projectile impact on a rigid wall in the target chamber. The camera recorded the phenomena with the frame rate of 1 × 10^6^ frames per second (FPS) at a resolution of 312 × 260 pixels. A polyvinylidene fluoride (PVDF) gauge (Piezo film stress gauge—PSG in Figure 4) was used to measure the transient pressure generated by the impacting projectile into a rigid wall. 

## 3. Experimental Results

**Figure 5:**
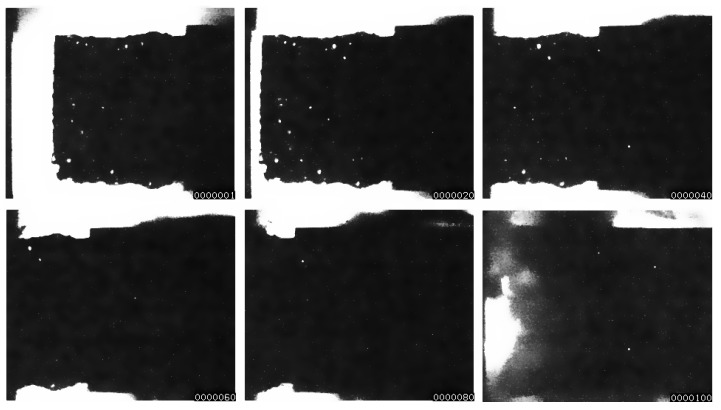
Impact sequence of the aluminum foam projectile hitting the rigid wall.

The high-speed recording sequences of projectile behavior before and upon impact into a rigid wall are shown in Figure 5, making it possible to analyze the deformation behavior of an open-cell aluminum foam sample. The images demonstrate that the aluminum foam samples deform only in the region contacting the rigid wall due to high impact velocity; the inertial effects dominate the deformation behavior. These experimental results confirmed the predictions of previous computer simulations of similar open-cell material behavior under impact loading conditions [20].

A more detailed analysis of the sample’s geometry before the impact (top left figure in Figure 5) shows that aluminum foam already plastically deforms while being accelerated through the barrel due to inertial effects caused by very high accelerations, which act on the specimen by combustion gases released immediately after the diaphragm rupture. The permanent initial deformation was estimated to be approximately 11.4%. The initial deformation was even higher during initial experiments, wherein more substantial quantities of explosives were used.

**Figure 6:**
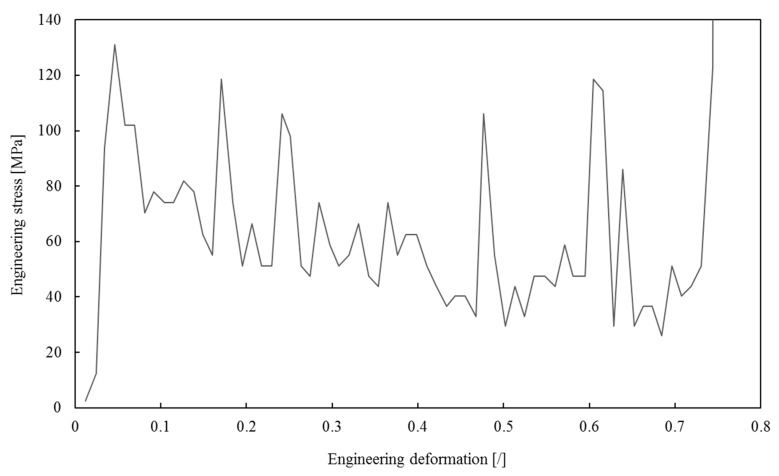
The computed engineering stress–strain relationship based on experimental measurements.

The pressure-time dependency, measured with a PSG sensor during the foam sample’s impact into the rigid wall, was used to compute the engineering stress–strain relationship (Figure 6), where the engineering stress represents a ratio between the computed impact force $F_{i}$ and the foam sample cross-section before the simulation $A_{0}$; i.e., $\sigma_{e}=F_{i}/A_{0}$. The engineering strain is calculated as$\;\varepsilon_{e}=\Delta h/h_{0}$, where Δh is the height change of the specimen and $h_{0}$ is the initial specimen height. The impact force was computed from the experimentally measured impact pressure and the surface of the stress gauge.

The engineering stress–strain relationship has a plateau-like shape during the high-velocity impact, during which inertial effects dominate the foam failure mechanism. After the initial elastic deformation, at which point the peak engineering stress equals 131 MPa, the plateau-like region follows until 71% strain is reached. The significant stress oscillations in this region are a consequence of discrete failures of subsequent foam layers with irregular structures. The average stress is slightly decreasing in this region, indicating a stress-softening behavior. The engineering stress rapidly increases after the 71% strain effect, indicating the foam densification.

## 4. Computer Simulations

Parametric computer simulations of the above described Taylor test were carried out to obtain a more detailed understanding of the deformation mechanism during the high-velocity impact test. All computer simulations were performed using the LS-DYNA explicit finite element code.

The initial deformation of the aluminum sample due to acceleration through the barrel was studied first. The acceleration of the sample during the Taylor impact test is highly non-linear. The maximum acceleration occurs immediately after the diaphragm rupture due to the sudden release of combustion gases. The actual acceleration of the sample through the barrel was impossible to measure in the experiments. A simple constant projectile acceleration variation was used in variational computer simulations to estimate the actual acceleration amplitude that causes the same averaged sample deformation at approximately 11.4% of the global strain, as observed in the experiments.

Parametric computational simulations of the foam sample’s impact into the rigid wall were performed next. The generated aluminum foam model was first accelerated to the experimentally-recorded impact velocity of 400 m/s and then impacted with the rigid wall. A reaction force on the rigid wall was computed and compared to the experimental data to validate the computational model.

### 4.1. Computational Modeling of Aluminum Foam with Irregular, Open-Cell Structure

A newly developed lattice-type model was used to model the irregular, open-cell aluminum foam samples. The model is based on the equilibrium liquid foam model with Weaire–Phelan cells [20,24]. The foam irregularity in the model was applied with controlled random displacements of Voronoi seed points of the equilibrium model:

(1) $$x_{k}=x_{k}^{i}+a\cdot d_{c}\cdot \phi_{k}$$

where$\;x_{k}^{i}$ (*k* ∈ [*x*, *y*, *z*]) are the original spatial coordinates of the seed point in the equilibrium model, a (∈ [0, 1]) is the irregularity parameter, $d_{c}$ is the representative cell size and $\phi_{k}$ (∈ [−1, 1]) is a random variable with uniform distribution. The representative cell size of generated computer models was $d_{c}=5$ mm, while the irregularity parameter was set to *a* = 0.2 [20].

The mesh vertices positions and their topology in Voronoi models represent the cell edges of the generated lattice models. A computational model of irregular, open-cell material is built from the lattice model, where each cell edge of the model is replaced with three to five Hughes–Liu type beam finite elements, depending on the length of the cell’s edge. The finite element nodes sharing the same lattice joint were merged into a single node [25].

The beam finite elements were assumed circular with a constant diameter along their length. The corresponding radius of the beam was calculated from the foam relative density $\rho_{r}$, the volume of the whole model V and the total length of all beam finite elements $l_{tot}$ as:

(2) $$r_{b}=\sqrt{\left(\rho_{r}\cdot V)/(l_{tot}\cdot \pi \right)}$$

and was equal to $r_{b}=0.60$ mm for all generated computer models in this study. The outer shape of the computational lattice model was cylindrical with the same dimensions as in samples used in experimental testing; i.e., the diameter of 35 mm and height of 35 mm.

The aluminum EN AW-1050 (99.5% purity) material properties were used in the computational simulations instead of the actual aluminum EN AW-1070 (99.7% purity), used to fabricate the foam samples. Both materials have very similar mechanical properties [26]. However, aluminum EN AW-1050 is more commonly used in the industry. Hence, its dynamic mechanical properties are readily available.

A bilinear elastoplastic constitutive model was used to model the base aluminum material in the computational model with the estimated density of $\rho_{al}=2700$ kg/m^3^, Young’s modulus E=70 GPa, the yield strength $\sigma_{y}=59$ MPa and the tangent modulus $E_{t}=84$ MPa, following measurements made by Berski et al. [27]. The Cowper–Symonds strain-rate dependency coefficients were C=6500 s^−1^ and p=4 [28].

### 4.2. Boundary Conditions

The lattice-type computational model of the irregular open-cell aluminum foam was supported by a rigid plate, representing the copper plate influence during the experiments. The plate was modeled by the shell-finite elements. The plate transferred loads of the combustion gases to the foam through a contact definition between the plate and the foam model. The contact conditions were also defined between the beam finite elements of the foam model to capture an essential mechanism of load transmission at larger deformations through the contact between the cell edges of the open-cell material. A penalty-based contact formulation was used for all described contact definitions. The rigid support plate was accelerated with a constant acceleration in the direction normal to the plate towards the foam model.

## 5. Computer Simulation Results

Many different aluminum foam sample models with randomly generated irregular lattice structures were generated first to obtain a representative statistical representation of the following simulations. 

**Figure 7:**
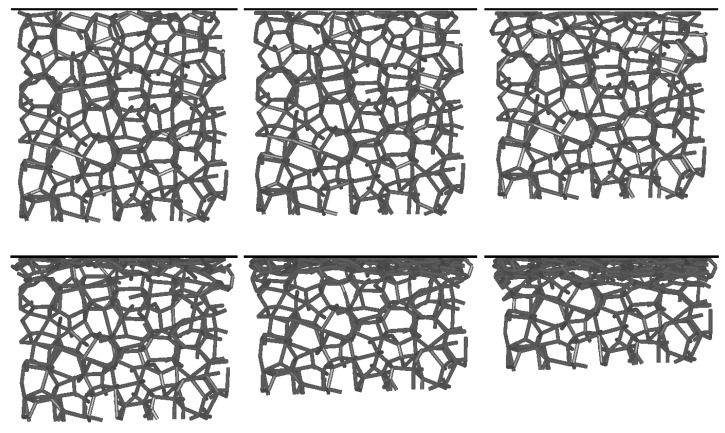
Deformation behavior of the foam lattice model at an acceleration of 3 × 10^5^ m/s^2^.

The effect of initial sample deformation due to acceleration in the bared was simulated first. Different foam sample models were used in twenty simulations, each at eleven different accelerations between 1 × 10^5^ and 9 × 10^5^ m/s^2^. The deformation behavior of a lattice foam model at 3 × 10^5^ m/s^2^ acceleration is shown in Figure 7. The images are rotated by 90 degrees so that the support plate is always above the foam model (the direction of the support plate motion is therefore downward) for easier comparison between different deformation stages. It can be seen that the deformation of the lattice foam model occurs only at the contact region with the support plate due to the distribution of inertia forces in the sample, which increases linearly towards the plate. The largest inertia force acts on the lattice layer closest to the support plate, causing its deformation. This is also the region with the most significant deformation, and is where the foam failure is initiated, which was also determined by other studies [29,30,31,32].

**Figure 8:**
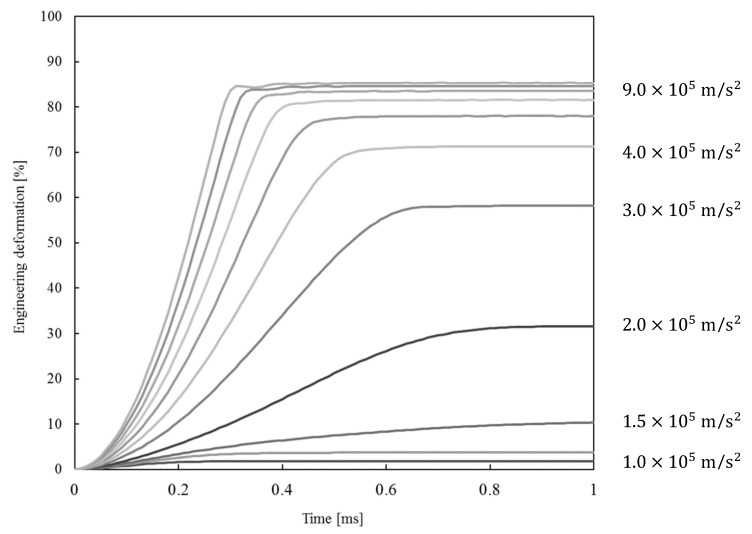
The effect of acceleration on the foam lattice model deformation.

The averaged engineering strain history of all computed samples at each acceleration level is shown in Figure 8. The specimen height change during simulations was calculated from the difference between the support plate position and the average nodal displacements on the foam surface opposite from the support plate. The simulation results show that the average deformation almost linearly increases in time up to a point, where the sample reaches a dynamic equilibrium. The average strains at the dynamic equilibrium range from 1.7% for the minimum acceleration up to 85.3% for the maximum acceleration. It was determined through interpolation that the acceleration of 1.52 × 10^5^ m/s^2^ of the foam lattice model is needed to achieve the initial specimen strain of 11.4%, as observed during the Taylor impact tests.

The computational simulations illustrate the very different deformation behavior of the open-cell foam at lower acceleration levels. In contrast, the differences become much smaller at higher accelerations. These differences can be attributed to the characteristic deformation behavior of the open-cell foam under compressive loading conditions. Namely, the open-cell materials reach a plateau stress region after the initial elastic and transition zone [14], where typically only a small increase in load (in our case, the acceleration) significantly increases the open-cell foam deformation. Further increase of the load causes quick densification of the open-cell foam, which contributes to smaller differences in deformation at higher accelerations.

The next set of parametric computational simulations was performed to study the foam sample’s impact on the rigid wall. The generated aluminum foam lattice model was first accelerated to the experimentally recorded impact velocity of 400 m/s and then impacted on the rigid wall, modeled by the shell finite elements with rigid material properties. The impact force was determined from reaction forces recorded at the rigid wall supports. Again, the impact of twenty generated foam lattice models with random irregular structures was computationally simulated to provide a better statistical representation of the results. 

**Figure 9:**
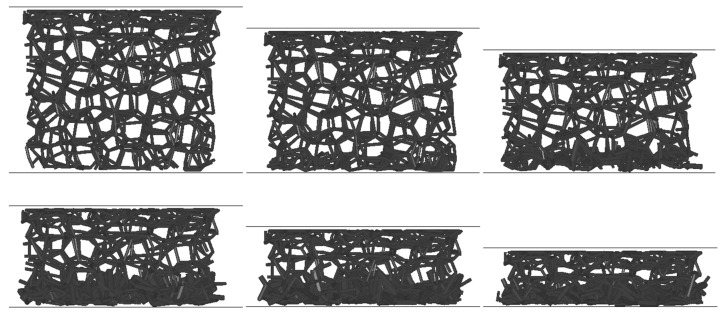
Deformation behavior of the foam lattice model’s impact into the rigid wall at the velocity of 400 m/s.

Figure 9 shows the deformation behavior of the foam lattice model during impact simulation. Only the middle internal vertical slice of the whole sample was plotted to improve the visibility of the results. The images of the deformation sequence were rotated in the same way as in Figure 7 (downward motion) for easier comparison. The initial deformation of the foam model at 11.4% strain at the contact with the support plate (top plate in Figure 9) was also considered. When the foam lattice model impacts the rigid-wall, the deformation of the open-cell foam starts at the contact with the support plate with failure of the first foam layer closest to the wall, again, due to the distribution of inertia forces, which are the highest right at the rigid wall. This behavior continues up to the full foam model compression.

**Figure 10:**
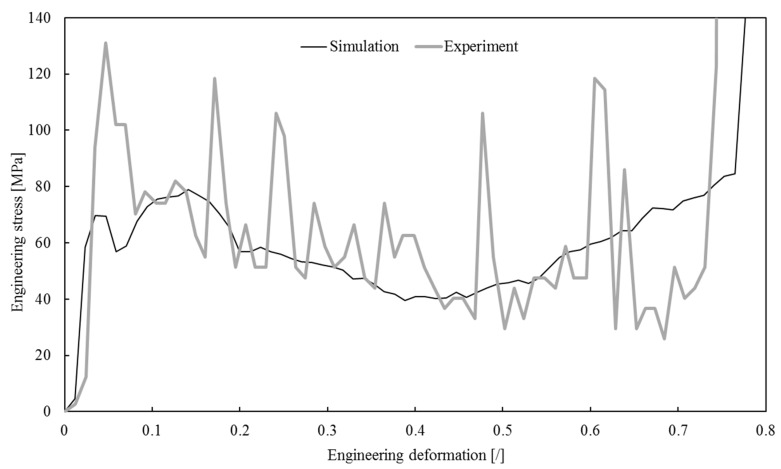
Experimental and computer simulated engineering stress–strain relationships.

Computed impact force-time distribution was used to derive the averaged engineering stress–strain relationship shown in Figure 10, together with the experimentally determined relationship.

### Comparison of Experimental and Computer Simulation Results

The comparison of experimental and simulation relationships in Figure 10 shows surprisingly good overall correspondence. The relationship obtained by simulations is much smoother due to the employed averaging process. The simulated relationship closely follows the shape of the experimental one up to the 65% strain. After that, the simulated stresses start to increase while the experimentally determined stresses continue to decrease until the strain of 70%. This increase in simulation stress is the result of the finite element formulation used. This formulation does not include the transverse deformation of the beam cross-sections, which leads to artificial stiffness increase when general beam to beam contact occurs close to foam sample densification strain. At the beginning of the deformation process, the number of such beam to beam contact pairs is small, and the stiffening is negligible. However, as the deformation increases, this becomes more and more prominent, as shown in the simulations.

Direct comparison of the engineering stress values between the computer simulation and experiment is difficult due to substantial oscillations in the experimental data, even after some filtering. Thus, a comparison of global strain energy densities was made to validate the computational foam lattice model. The global strain energy densities were computed from the engineering stress–strain relationship up to the 70% strain by applying the Newton–Cotes formula with the trapezoidal rule. The computed strain energy densities from computational simulations and the experimental observations are equal to and 41.09 MJ/m^3^, respectively. The relative difference of 7.1% is relatively small for this type of a problem, especially considering substantial oscillations in the experimental data and limitations of the beam finite element formulation. It can be concluded that the computer model appropriately represents the impact conditions of open-cell foams and can be recommended for application in similar high-velocity impact investigations.

## 6. Conclusions

The study presents the results of the experimental and computational study of the high-velocity impact of low-density aluminum foam. Previously conducted experimental Taylor impact tests showed that the studied aluminum foam samples deform due to inertial effects when accelerated to the impact speed of 400 m/s through the barrel of the testing device. The aluminum foam samples deform only in the region contacting the rigid wall during impact, due to the high impact velocity; the inertial effects dominate the deformation process. Despite this, the engineering stress–strain relationship retains its plateau shape until the densification strain, typical for cellular materials. 

The experimental tests were successfully reproduced with parametric computer simulations using the LS-DYNA explicit finite element code. They provided better insight into the foam deformation process during the high-velocity impact test. An exclusive computational lattice-type model was used for this purpose, which can reproduce the randomness of the irregular open-cell structure of aluminum foams. Parametric computer simulations of twenty different aluminum foam sample models with randomly generated irregular lattice structures at different accelerations were carried out to obtain representative statistical results. Computer simulations have shown that the acceleration of approximately 1.52 × 10^5^ m/s^2^ is needed to deform the aluminum foam sample to the same engineering deformation as observed during the experiment. The simulations also showed that the material deforms only in the vicinity of the acceleration plate due to the distribution of significant mass inertia forces at high acceleration. The high strain-rate sensitivity of low-density aluminum foam was also observed. A comparison of experimental and computational results during aluminum foam sample impact shows very similar deformation behavior. The correlation of computationally calculated and experimentally measured strain energy densities is excellent. The computer model of low-density aluminum foam impact at high speed correctly represents the real impact conditions and can be recommended for use in similar high-velocity impact investigations.
